# Letter to the editor: “A systematic review and network meta-analysis comparing upfront surgery with neoadjuvant treatments in pancreatic cancer”

**DOI:** 10.1097/JS9.0000000000002226

**Published:** 2025-01-09

**Authors:** Qiang Yi, Gangfeng Zhu, Jinghua Zhong

**Affiliations:** a The First Clinical Medical College, Gannan Medical University, Ganzhou, Jiangxi Province, China; b Department of Oncology, The First Affiliated Hospital of Gannan Medical University, Ganzhou, Jiangxi Province, China

*Dear Editor,*

We have read with interest the study by He *et al*. In this study, the authors aimed to explore optimal neoadjuvant treatment strategies for resectable, borderline resectable, and locally advanced pancreatic cancers, providing a theoretical basis for clinical practice. The study involved 889 patients who underwent treatment methods, including upfront surgery, neoadjuvant chemotherapy, and preoperative neoadjuvant chemoradiotherapy. Network meta-analysis results demonstrated that compared to upfront surgery and neoadjuvant chemotherapy, surgery after neoadjuvant chemoradiotherapy effectively improved overall survival in patients with resectable and borderline resectable pancreatic cancer (RPC) and significantly increased the rate of R0 resection and pathologically negative lymph nodes (pN0). However, neoadjuvant chemoradiotherapy increased the risk of grade ≥ 3 treatment-related adverse events, even in patients with locally advanced pancreatic cancer^[1]^.

Despite these clear results, there are some concerns regarding the study’s statistical findings.

First, sensitivity analysis is crucial for assessing the reliability of the results by examining the effect of different hypotheses, datasets, and analytical methods on the final outcomes. Although the authors mentioned conducting a sensitivity analysis to explore the sources of heterogeneity, this analysis was not implemented in the manuscript, thereby weakening the credibility and interpretability of the results. This omission complicates the understanding and generalization of the findings and may affect the development of clinical applications and patient management strategies.

Second, although the authors referenced the use of funnel plots to assess publication bias, this method may lack sensitivity in small sample sizes, potentially failing to accurately reflect the true situation of publication bias. We recommend combining it with other statistical methods, such as Egger’s test or the trim-and-fill method, for a more comprehensive evaluation of publication bias.

Finally, although the authors employed *Q* tests and *I*^2^ statistics to assess heterogeneity, the background for interpreting *I*^2^ values is insufficient. Typically, *I*^2^ < 25% indicates low heterogeneity, whereas 50% indicates moderate heterogeneity, which may lead to ambiguity in the interpretation of the study results. Furthermore, although the diagnostic criteria for RPC, BRPC, and LAPC were mentioned, the specific methods used to confirm these diagnoses (imaging or pathological criteria) were not detailed, potentially introducing subjectivity into the inclusion criteria. The lack of detailed descriptions regarding specific protocols for neoadjuvant chemoradiotherapy, neoadjuvant chemotherapy, and upfront surgery (dosage, cycles, and treatment duration) fails to consider confounding factors that are crucial for the comparison and interpretation of results.

Nevertheless, despite the necessity to address these points, we sincerely appreciate the authors for providing insights into optimal neoadjuvant treatment strategies for advanced pancreatic cancer and opportunities to formulate new neoadjuvant treatment protocols in clinical practice.

## Data Availability

This is a comment on apublished paper; no data was presented in our comment, so the data statement is not applicable.
